# Supplementary material for: Decreased Passive Immunity to Respiratory Viruses through Human Milk during the COVID-19 Pandemic
Source: Microbiol Spectr. 2022 Jun 28;10(4):e00405-22. doi: 10.1128/spectrum.00405-22 (PMC9431045; doi:10.1128/spectrum.00405-22)
Supplement: Supplemental file 1 — Fig. S1 to S3. Download spectrum.00405-22-s0001.pdf, PDF file, 0.7 MB [file spectrum.00405-22-s0001.pdf]

Supplementary figures

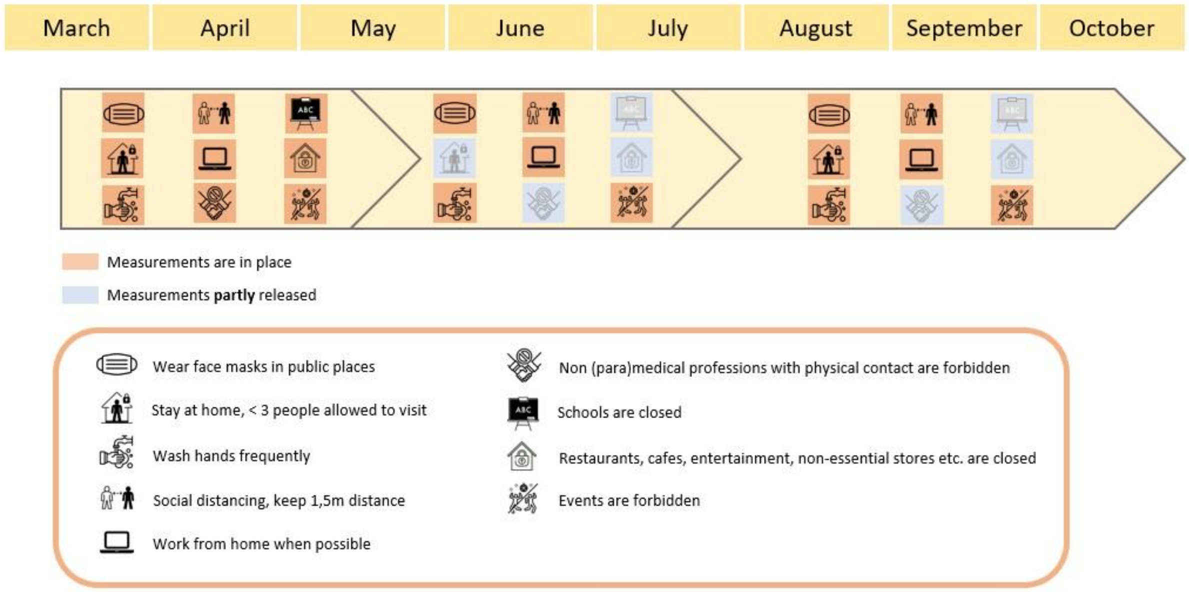

Supplementary Figure 1. Preventative measures in the Netherlands during the study period.

Overview of the national preventative measures in the Netherlands during the study period, orange indicates that the measure was in place during that period, and grey indicates that the measure was partially released.

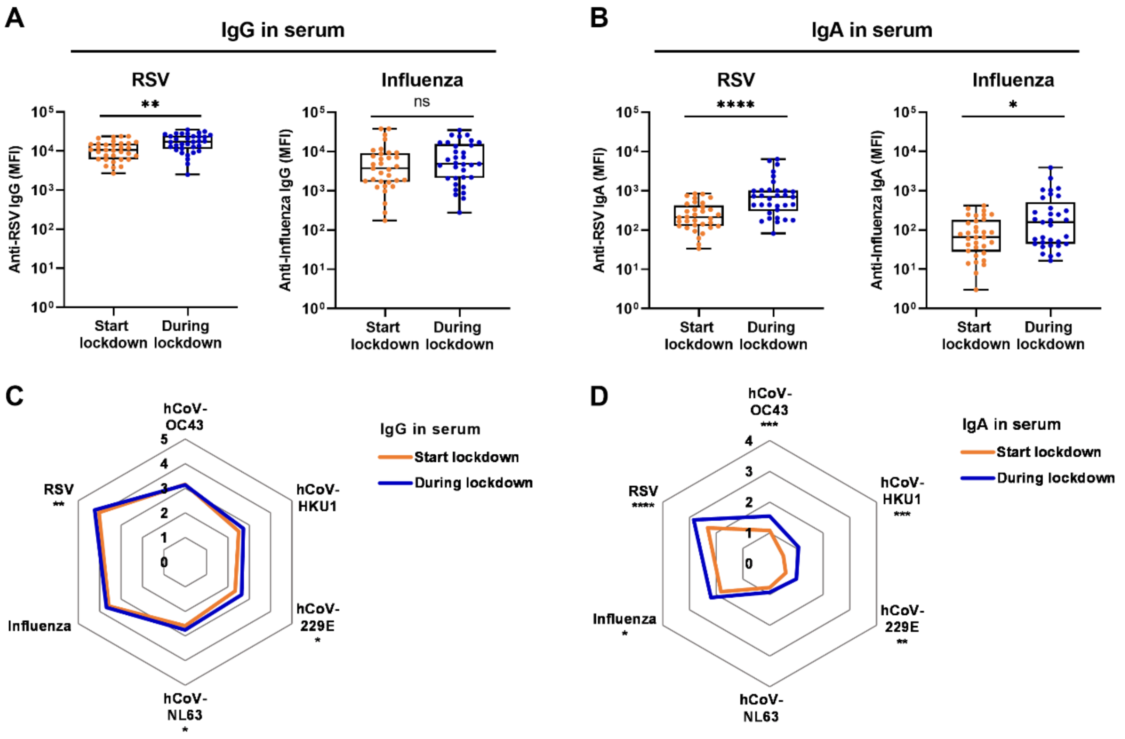

Supplementary Figure 2. Levels of respiratory virus antibodies in serum of lactating mothers at the start of the pandemic and during the pandemic.

(A) Anti-RSV (left) and anti-Influenza (right) IgA levels in mean fluorescence intensity (MFI) in serum of 34 mothers at the start of the COVID-19 lockdown (April-May 2020, orange dots), compared to 34 mothers during the COVID-19 lockdown (October-November 2020, blue dots) using a Mann-Whitney U test. Boxplots range the minimum and maximum values. *** = p <0.001; ** = p < 0.01. (B) Anti-RSV (left) and anti-Influenza (right) IgA levels in human milk of 46 mothers at the start of the COVID-19 lockdown (April-May 2020, orange dots) were compared to 34 mothers during the COVID-19 lockdown (October-November 2020, blue dots) using a Mann-Whitney U test. (C) Spiderweb plot showing IgG levels and (D) IgA levels to all viral antigens as the logarithm of the MFI of each group. The two groups were compared individually per antigen with a Mann-Whitney U test. *** = p < 0.001; ** = p < 0.01; * = p < 0.05; ns or no asterisk = not significant.

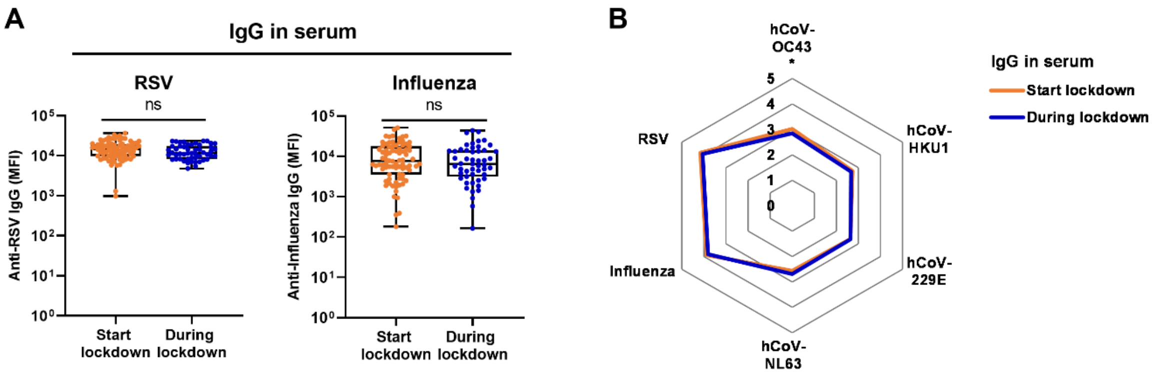

Supplementary Figure 3. Respiratory virus antibody levels in serum were assessed for six months from the start of the first COVID-19 lockdown.

(A) Anti-RSV (left) and anti-Influenza (right) IgG levels in mean fluorescence intensity (MFI) in serum of 82 COVID-19 negative individuals at the start of the COVID-19 lockdown (April - May 2020, orange dots), compared to 51 COVID-19 negative individuals during the COVID-19 lockdown (October-November 2020, blue dots) using a Linear Mixed Models analysis. (B) Spiderweb plot showing IgG levels to all viral antigens as the logarithm of the MFI of each group. The two groups were compared individually per antigen with a Linear Mixed Models analysis. * = $p < 0.05$; ns = not significant
